# Prevalence and steatosis severity of MASLD in patients with type 2 diabetes mellitus in Kuwait

**DOI:** 10.3389/fendo.2026.1963647

**Published:** 2026-10-13

**Authors:** Munira Altarrah, Rashed Alhammad, Saoud Ahmad, Mohamed Abu-Farha, Sriraman Devarajan, Shaymaa Alhaddad, Abeer Afyoni, Fouz AlHaifi, Mohamed Qaddoumi, Mohammad Shehab, Mohamed Salah, Hamad Ali, Muhammed Abdul Ghani, Fahd Al-Mulla, Fahad Alajmi, Jehad Abubaker

**Affiliations:** 1 Unit of Gastroenterology and Transplant Hepatology, Thunayan Al Ghanim Gastroenterology Center, Al Amiri Hospital, Kuwait City, Kuwait; 2 Department of Pharmacology, Faculty of Medicine, Kuwait University, Kuwait City, Kuwait; 3 Department of Translational Research, Dasman Diabetes Institute, Kuwait City, Kuwait; 4 Department of Biochemistry and Molecular Biology, Dasman Diabetes Institute,Kuwait City, Kuwait; 5 National Dasman Diabetes Biobank, Dasman Diabetes Institute, Kuwait City, Kuwait; 6 Department of Pharmacology, Faculty of Pharmacy, Kuwait University, Kuwait City, Kuwait; 7 Program of Medicine, College of Medicine and Health Sciences, Abdullah Al Salem University, Khaldiya, Kuwait; 8 Department of Medical Laboratory Sciences, Faculty of Allied Health Sciences, Health Sciences Center, Kuwait University, Jabriya, Kuwait; 9 Division of Diabetes, University of Texas Health Science Center, San Antonio, TX, United States

**Keywords:** fatty liver, MASLD, metabolic disease, steatosis, type 2 diabetes

## Abstract

**Background:**

Metabolic dysfunction–associated steatotic liver disease (MASLD) is a common comorbidity in individuals with type 2 diabetes mellitus (T2DM). Given the high prevalence of T2DM in Kuwait and limited regional data on hepatic steatosis, this study aimed to assess the prevalence and severity of liver steatosis among T2DM patients attending Dasman Diabetes Institute (DDI).

**Methods:**

A total of 267 T2DM patients (mean age, 53.2 ± 9.8 years; 67.4% men and 32.6% women) were evaluated using vibration-controlled transient elastography (VCTE; FibroScan) to determine liver steatosis through controlled attenuation parameter (CAP) scores. MASLD was classified based on CAP thresholds: S0 (<238 dB/m), S1 (238–259 dB/m), S2 (260–290 dB/m), and S3 (>290 dB/m). Liver stiffness measurement (LSM) was used to estimate fibrosis severity.

**Results:**

Hepatic steatosis (S1–S3) was present in 72.7% (n = 194) of participants, with 40.8% (n = 109) exhibiting severe steatosis (S3), 20.6% (n = 55) moderate steatosis (S2), and 11.2% (n = 30) mild steatosis (S1). Minimal or no fibrosis (F0–F1) was present in 92.5% (n = 247), while moderate fibrosis (F2) was present in 4.1% (n=11), and advanced fibrosis (F3–F4) was identified in 3.4% (n = 9) of the cohort. CAP scores showed significant positive associations with BMI (ρ = 0.32, p < 0.001), waist circumference (ρ = 0.200, p = 0.001), triglycerides (ρ = 0.310, p < 0.001), ALT (ρ = 0.325, p < 0.001), AST (ρ = 0.197, p = 0.002), and GGT (ρ = 0.203, p = 0.001), while an inverse correlation was observed with HDL-C (ρ = −0.19, p = 0.003). In multivariable linear regression analyses, CAP score remained independently associated with BMI, ALT, and LSM. Conversely, LSM was associated significantly with CAP score, BMI, ALT, AST, and GGT.

**Conclusion:**

In this studied Kuwaiti cohort, MASLD was present in over 70% of adults with T2DM, with a substantial subset exhibiting advanced steatosis. The severity of both steatosis and fibrosis showed significant associations with metabolic and hepatic biomarkers, reinforcing their pathophysiological linkage. These findings support the integration of standardized liver disease risk stratification into routine diabetes care, particularly in populations at elevated cardiometabolic risk.

## 1 Introduction

Metabolic dysfunction–associated steatotic liver disease (MASLD), previously included under non-alcoholic fatty liver disease (NAFLD), is among the most prevalent chronic liver diseases worldwide. It is defined by the presence of hepatic steatosis, characterized by excessive accumulation of triglycerides within the liver, in conjunction with metabolic dysfunction (1). The diagnosis of MASLD is based on the presence of objectively confirmed hepatic steatosis together with metabolic risk factors, including overweight or obesity (BMI > 25kg/m2), type 2 diabetes mellitus (T2DM), or other metabolic dysregulation such as prediabetes, low High-Density Lipoprotein cholesterol (HDL-C), and hypertriglyceridemia (1, 2). MASLD has emerged as the most prevalent form of chronic liver disease among individuals with T2DM (3). The interplay between MASLD and T2DM is bidirectional, driven by shared mechanisms including insulin resistance, persistent low-grade inflammation, and broader metabolic dysfunction (2, 3).

MASLD has a global prevalence of approximately 32.4%, with higher rates in males (39.7%) than in females (32.0%) (4). Considerable racial, ethnic, and geographic variations exist, with the Middle East and North Africa (MENA) region exhibiting some of the highest reported prevalence rates at 36.5%, affecting roughly 162 million people, representing the second-highest global burden after Latin America (4, 5). Kuwait bears one of the highest burdens of MASLD globally. A recent systematic review and meta-regression analysis of the MENA region estimated the prevalence of NAFLD/MASLD in Kuwait at 45.37%, among the highest country-specific estimates in the region, alongside Egypt and Qatar (6, 7). The clinical consequences of this burden are already becoming apparent, Kuwaiti data from 2019 revealed that 63% of patients referred for liver transplantation in 2018–2019 had NASH-related cirrhosis (8), underscoring the substantial burden of advanced metabolic liver disease.

Insulin resistance and MASLD are closely interconnected, as reflected by their high co-prevalence and shared pathophysiological mechanisms. Globally, over 436 million adults are currently living with T2DM, and recent meta-analysis estimate that approximately 65% of these individuals have MASLD, with reported prevalence ranging from ~59% to over 72% depending on region (9). Epidemiological data indicate that the prevalence of MASLD among T2DM patients has increased steadily over recent decades, from 55.9% in the early 2000s to 68.8% between 2016 and 2021 (10). The prevalence of MASLD among T2DM patients continues to rise, underscoring the increasing hepatic burden. Among those with T2DM, the prevalence of the more severe form, Metabolic Dysfunction-Associated Steatohepatitis (MASH), is estimated to be around 35-46.88% (9). Disease progression within this population is substantial, among those with MASLD, 66% progress to metabolic steatohepatitis (MASH), 41% exhibit significant fibrosis, and 15% develop advanced fibrosis or cirrhosis (11, 12). This global burden is particularly pronounced Kuwait, which has one of the highest rates of T2DM worldwide, with a prevalence of 25.6%, accounting for approximately 908,500 individuals, placing Kuwait among the countries with greatest metabolic disease burden, and consequently, among those most vulnerable to the hepatic complications. Collectively, these data underscore the substantial and growing hepatic disease burden among individuals with T2DM and reinforce the need for systematic liver disease screening and risk stratification, particularly in high-risk populations.

Despite the high prevalence of T2DM and metabolic risk factors in Kuwait and the broader MENA region, there is limited data characterizing the burden and severity of MASLD. The asymptomatic nature of early-stage MASLD, coupled with its potential progression to advanced fibrosis, highlights the need for region-specific evidence to guide local screening and management strategies. Therefore, this cross-sectional study was conducted to assess the prevalence and severity of hepatic steatosis in 267 adults with T2DM who were attending the Dasman Diabetes Institute (DDI) in Kuwait.

## 2 Materials and methods

### 2.1 Study design and participants

This cross-sectional, single-center study initially included 274 adults with a confirmed diagnosis of T2DM attending the outpatient clinics of Dasman Diabetes Institute (DDI), a specialized diabetes center in Kuwait providing clinical care for individuals with diabetes and related metabolic disorders. Participants were recruited from patients attending DDI outpatient clinics and therefore represent a clinical T2DM cohort rather than a population-based, nationally representative sample of individuals with T2DM in Kuwait. All participants provided written informed consent prior to enrollment. All participants provided written informed consent prior to their enrolment. Exclusion criteria included: (1) history of viral hepatitis (HBV or HCV), (2) significant alcohol consumption (3) autoimmune liver disease, (4) use of hepatotoxic or steatogenic medications, and (5) other known causes of secondary fatty liver. Seven participants were excluded due to invalid or incomplete FibroScan CAP or liver stiffness measurements, resulting in a final analytic sample of 267 participants. The study was approved by the Ethical Review Committee of the DDI and conducted in accordance with the principles of the Declaration of Helsinki.

### 2.2 Clinical and anthropometric assessment

Demographic and clinical information, including gender, age, and diabetes duration, were obtained using structured questionnaires and medical records. Anthropometric measurements were taken by trained staff following standardized protocols. Height and weight were measured to the nearest 0.1 cm and 0.1 kg, respectively, and BMI was calculated as weight (kg) divided by height squared (m²). Waist circumference was measured midway between the lowest rib and the iliac crest at the end of normal expiration. Blood pressure [systolic (SBP) and diastolic (DBP)] was recorded in the seated position after a 5-minute rest using an automated sphygmomanometer; the mean of two consecutive readings served as the final measure.

### 2.3 Biochemical measurements

Fasting venous blood samples were collected after an overnight fast of 8–12 hours. The parameters measured included fasting glucose, lipid profile [i.e. total cholesterol (TC), triglycerides (TG), HDL-C, liver enzymes [i.e. alanine aminotransferase (ALT), aspartate aminotransferase (AST), and gamma-glutamyl transferase (GGT)], and serum creatinine. Platelet counts were obtained using an automated hematology analyzer (Sysmex XE-5000, Kobe, Japan). Low-density lipoprotein cholesterol (LDL-C) was calculated using the Friedewald equation rather than measured directly: LDL-C (mmol/L) = TC − HDL-C − (TG/2.2), where TC represents total cholesterol, HDL-C represents high-density lipoprotein cholesterol, and TG represents triglycerides.

### 2.4 MASLD and T2DM assessment

T2DM was diagnosed in accordance with the American Diabetes Association (ADA) 2023 criteria, based on established diagnostic glucose thresholds, including fasting plasma glucose ≥7.0 mmol/L, 2-hour plasma glucose ≥11.1 mmol/L, or random plasma glucose ≥11.1 mmol/L in the presence of symptoms of hyperglycemia. MASLD was defined in accordance with the 2023 multi-society Delphi consensus as the presence of hepatic steatosis together with at least one cardiometabolic risk factor. In the present study, hepatic steatosis was assessed using the FibroScan-derived controlled attenuation parameter (CAP). Relevant cardiometabolic risk factors included overweight/obesity, T2DM or other glycemic dysregulation, elevated triglycerides, and low high-density lipoprotein cholesterol (HDL-C). As all participants in the present cohort had established T2DM, participants with CAP-defined hepatic steatosis fulfilled the cardiometabolic risk component of the MASLD definition.

### 2.5 Liver fat and fibrosis assessment

The primary outcome variable was the Controlled Attenuation Parameter (CAP) score, which reflects hepatic steatosis severity. Based on the established steatosis grading system, CAP values were categorized as follows: S0 (no steatosis, <238 dB/m), S1 (mild steatosis, 238–259 dB/m), S2 (moderate steatosis, 260–290 dB/m), and S3 (severe steatosis, >290 dB/m). Liver stiffness measurement (LSM, expressed in kilopascals [kPa]) was used to estimate fibrosis severity and categorized as follows: F0–F1 (no or mild fibrosis) 2.0–7.5 kPa, F2 (moderate fibrosis) 7.5–10.0 kPa, F3 (advanced fibrosis) 10.0–14.0 kPa, and F4 (cirrhosis) ≥14.0 kPa, in accordance with commonly used thresholds in MASLD populations.

### 2.6 Statistical analysis

Descriptive statistics are reported as mean ± standard deviation (SD), or median (interquartile range [IQR]) for continuous variables, depending on underlying data distribution. Categorical variables are summarized as absolute frequencies (percentages) and compared using Fisher’s exact test or the Chi-square χ2 test. Normality of data distributions was formally evaluated using the Shapiro-Wilk test. For univariate comparisons of continuous baseline clinical and biochemical variables across hepatic steatosis groups (S0 to S3), one-way analysis of variance (ANOVA) was applied for normally distributed parametric metrics. The non-parametric Kruskal-Wallis H test was utilized when data distributions violated normality assumptions, followed by Dunn’s post-hoc test with alpha adjustments to evaluate pairwise differences between specific steatosis tiers. Unadjusted bivariate associations between continuous liver fat measure (Controlled Attenuation Parameter [CAP] score) and independent anthropometric or metabolic markers were examined using Spearman’s rank correlation coefficient (ρ). This univariate comparisons, corresponding post-hoc analyses, and high-resolution box plots were executed and generated using GraphPad Prism software (version 9.0, GraphPad Software, Boston, MA, USA). To control demographic and clinical confounding factors, partial Spearman’s rank correlation analyses adjusting for age and BMI were conducted. Subsequently, two separate multivariable linear regression models were constructed to evaluate independent clinical predictors of hepatic disease. For Model 1, the continuous steatosis CAP score was established as the dependent variable, with liver stiffness measurement (LSM, kPa), age, BMI, ALT, AST, and GGT serving as independent predictors. For Model 2, LSM was designated as the dependent variable against the same cohort of clinical and metabolic covariates. Prior to building the final regression models, necessary assumption regarding linearity, normality of residuals, homoscedasticity, and multicollinearity (evaluated via Variance Inflation Factors [VIF]) were verified. Regression results are expressed as unstandardized coefficients (β) with their corresponding standard errors (SE), 95% confidence intervals (95% CI), and exact p-values. All multivariable correlation and linear regression modeling workflows were carried out using IBM SPSS Statistics software (version 25.0, IBM Corp., Armonk, NY, USA). For all analyses, a two-tailed p-value < 0.05 was considered statistically significant. To assess potential confounding by diabetes pharmacotherapy, additional multivariable linear regression analyses were performed incorporating available treatment information. Treatment was categorized as insulin therapy, metformin monotherapy, or multidrug therapy, with multidrug therapy representing combined glucose-lowering regimens. These treatment variables were included alongside age, BMI, liver enzymes (ALT, AST, and GGT), and either LSM or CAP, as appropriate. Multicollinearity was assessed using variance inflation factors (VIFs), which were below 3.8 for all predictors. Detailed treatment distributions and treatment-adjusted regression models are presented in **Supplementary Tables 1**, **2**.

### 2.7 Sample size and power consideration

A minimum sample size of 200 participants was estimated to provide >80% statistical power to detect a moderate effect size (Cohen’s d = 0.4) in key metabolic parameters (e.g., BMI, ALT, and triglycerides) between T2DM participants with and without fatty liver, at an alpha level of 0.05. The final sample of 267 participants (73, [27.3%] with no evidence of steatosis ([S0]) and 194 [72.7%] exhibiting steatosis ([S1-S3]) exceeded the minimum requirement, ensuring sufficient statistical power for subgroup comparisons.

## 3 Results

### 3.1 FibroScan CAP grading and cohort distribution

A total of 274 subjects with diagnosed T2DM were recruited for the study. Participants without valid CAP or liver stiffness measurements were excluded, resulting in 267 participants included in the final analysis. Based on FibroScan CAP grading, the prevalence of hepatic steatosis was high, with 72.7% of participants classified as having steatosis (**Table 1**). Severe steatosis (S3) was present in 40.8% of the cohort, whereas 27.3% showed no evidence of steatosis (S0). The number of participants with liver steatosis across all groups is indicated in **Figure 1A**, in which there are 73 participants with no evidence of steatosis (S0), 30 participants with mild steatosis (S1), 55 participants with moderate steatosis (S2), and 109 participants with severe steatosis (S3) **Figure 1B**.

**Table 1:** Prevalence of hepatic steatosis (CAP) in T2DM patients in Kuwait.

Outcome	Definition	n/N	Prevalence (%)	95% CI	Notes
Any steatosis	S1–S3 (CAP ≥238 dB/m)	194/267	72.7	66.9–77.8	Primary estimate
Severe steatosis	S3 (CAP >290 dB/m)	109/267	40.8	35.1–46.7	High-risk subgroup
S0 (no steatosis)	CAP <238 dB/m	73/267	27.3	22.2–33.1	
S1 (mild)	CAP 238–259 dB/m	30/267	11.2	7.9–15.6	
S2 (moderate)	CAP 260–290 dB/m	55/267	20.7	16.2–26.1	
S3 (severe)	CAP >290 dB/m	109/267	40.8	35.1–46.7	

CAP cutoffs: S0 <238 dB/m; S1 238–259 dB/m; S2 260–290 dB/m; S3 >290 dB/m. Prevalence estimates use 95% confidence intervals (Wilson method).

**Figure 1:**
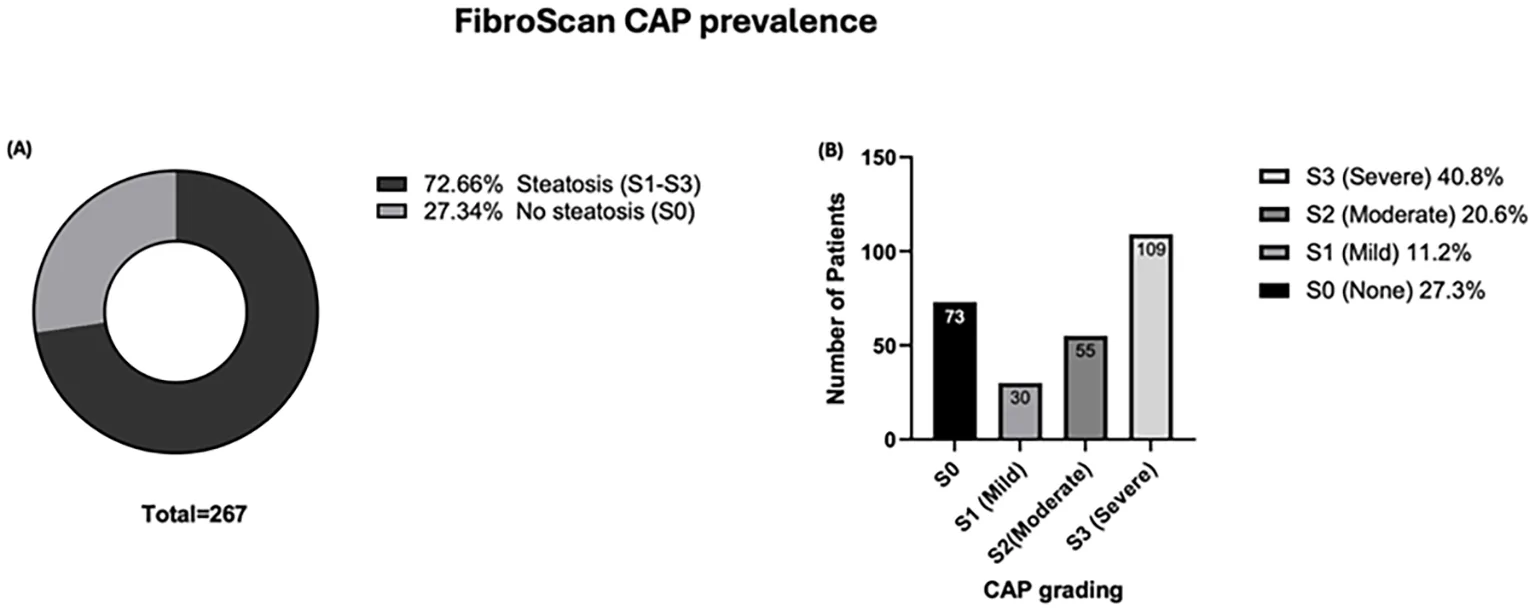
Prevalence and distribution of hepatic steatosis grades assessed by CAP in patients with T2DM (n=267). **(A)** Donut chart illustrating the overall prevalence of hepatic steatosis, with 72.7% of patients classified as having steatosis (S1-S3) and 27.3% showing no evidence of steatosis (S0). **(B)** Bar chart showing the distribution of participants across individual steatosis grades: S0 (no steatosis, CAP <238 dB/m, n=73, 27.3%), S1 (mild steatosis, CAP 238–259 dB/m, n=30, 11.2%), S2 (moderate steatosis, CAP 260-290 dB/m, n=55, 20.6%), S3 (severe steatosis, CAP > 290 dB/m, n=109, 40.8%). Numbers above bars indicate participant counts.

Median age of participants across hepatic steatosis groups was compared. Participants without steatosis (S0) had a median age of 54 years, while median age in the mild (S1), moderate (S2), and severe (S3) steatosis groups were 54, 55, and 53 years, respectively. A statistically significant difference in median age was observed across groups (P = 0.044; **Table 1**).

### 3.2 Clinical and metabolic characteristics stratified by steatosis grade

Clinical and biochemical characteristics stratified by steatosis severity are presented in **Table 2** and **Figures 2A–H**. BMI and waist circumference increased significantly across steatosis grades (both p<0.001; **Figures 2A, B**), with post-hoc analysis confirming significant differences between S0 and S3, S1 and S3 (both p<0.0001). SBP increased progressively from 125 mmHg to 134 mmHg in S3 (p=0.015; **Figure 2C**). DBP also differed significantly across groups, with a significant difference between S0 and S3 (p<0.05; **Figure 2D**); however, no consistent directional trend was observed. AST showed a modest but significant overall difference across steatosis grades (p=0.013; **Figure 2E**), though no individual pairwise comparison reached significance, with the elevation confined to the S3 group (median 22 vs 19 U/L in S0-S2). ALT and GGT were significantly elevated across groups (both p<0.001; **Figures 2F, G**), driven by markedly higher values in S3. Triglycerides showed a consistent graded increase from 0.88 mmol/L in S0 to 1.69 mmol/L in S3 (p<0.001; **Figure 2H**). HbA1c, LDL-C, total cholesterol, creatinine, and platelet count did not differ significantly (all p>0.05).

**Table 2:** Median comparison of fibro scan steatosis CAP score with clinical parameters.

Parameters	*S0* (no steatosis, <238 dB/m)	*S1* (mild steatosis, 238–259 dB/m)	*S2* (moderate steatosis, 260–290 dB/m)	*S3* (severe steatosis, >290 dB/m)	P-value
Number	73 (27.3%)	30 (11.2%)	55 (20.6%)	109 (40.8%)	
Gender (M% F%) ***	65.8%/34.2%	80.0%/20.0%	61.8%/38.2%	68.8%/31.2%	0.349
Age (in years), median (IQR)*	54.00 (40.50,60.00)	54.00 (47.25,65.25)	55.00 (51.00,63.00)	53.00 (45.50,58.00)	0.044
Height (cms), Mean ± SD**	168.00 ± 9.44	170.02 ± 9.03	167.74 ± 9.79	169.78 ± 9.83	0.431
Weight (Kgs), Mean ± SD**	78.98 ± 18.39	80.82 ± 16.43	85.36 ± 15.16	94.82 ± 19.00	<0.001
BMI, kg/m^2^ median (IQR)*	27.35 (24.13,29.91)	26.88 (24.77,30.45)	30.71 (26.18,32.81)	31.94 (29.12,35.73)	<0.001
Waist Cir. (cm) Mean ± SD**	95.13 ± 14.32	97.93 ± 10.38	102.82 ± 11.72	108.20 ± 12.81	<0.001
SBP, mmHg, median (IQR)*	125.00 (115.00,136.00)	128.00 (123.25,139.50)	129.00 (119.00,140.00)	134.00 (123.50,143.50)	0.014
DBP, mmHg, median (IQR)*	78.00 (70.00,86.00)	83.50 (73.00,91.50)	78.00 (70.00,89.00)	82.00 (75.00,89.00)	0.012
S.Creat., umol/L, median (IQR)*	72.00 (56.50,82.00)	79.50 (66.00,84.50)	71.00 (60.00,86.00)	73.00 (61.00,87.50)	0.226
ALT, U/L, median (IQR)*	28.00 (22.00,39.50)	31.00 (27.75,34.50)	31.00 (27.00,38.00)	38.00 (29.00,55.00)	<0.001
AST, U/L, median (IQR)*	19.00 (15.50,23.50)	19.00 (16.00,21.25)	19.00 (15.00,22.00)	22.00 (17.00,30.00)	0.013
GGT, U/L, median (IQR)*	28.00 (21.00,40.50)	31.00 (22.00,43.25)	30.00 (25.00,43.00)	38.00 (32.00,52.00)	<0.001
TC, mmol/L, Mean ± SD **	4.63 ± 1.28	4.67 ± 1.30	5.00 ± 1.29	4.79 ± 1.27	0.180
TG, mmol/L, median (IQR)*	0.88 (0.60,1.60)	1.20 (0.93,1.66)	1.45 (1.01,2.12)	1.69 (1.14,2.30)	<0.001
HDL-C, mmol/L, median (IQR)*	1.42 (1.14,1.70)	1.36 (1.08,1.63)	1.29 (1.09,1.59)	1.22 (1.02,1.39)	0.006
LDL-C, mmol/L, median (IQR)*	2.60 (1.83,3.48)	2.65 (1.50,3.55)	2.50 (1.70,3.30)	2.90 (2.00,3.60)	0.228
HbA1C, %, median (IQR)*	6.55 (5.63,7.53)	6.65 (5.80,7.20)	6.80 (6.08,7.43)	7.00 (6.20,8.60)	0.070
Platelet x 10^9^/L, median (IQR)*	267.00 (221.00,293.00)	256.00 (210.25,288.25)	273.00 (232.00,311.00)	271.00 (232.00,311.50)	0.467
LSM kPa, median (IQR)*	4.50 (3.70,5.40)	4.40 (3.88,4.80)	4.50 (4.00,5.10)	5.20 (4.45,6.20)	<0.001

IQR, Inter quartile range; BMI, Body Mass Index; SBP, Systolic Blood Pressure; DBP, Diastolic Blood Pressure; S.Creat., Serum Creatinine; ALT, Alanine Transaminase; AST, Aspartate aminotransferase; GGT, gamma-glutamyl transferase; TC, Total Cholesterol; TGL, Triglycerides; HDL-C; high-density lipoprotein cholesterol; LDL-C, low-density lipoprotein cholesterol, CAP, Controlled Attenuation Parameter. *P-value continuous variable non-normal using Kruskal Wallis Test, **P-Value using parametric One-Way ANOVA, ***Fisher’s exact Test.

**Figure 2:**
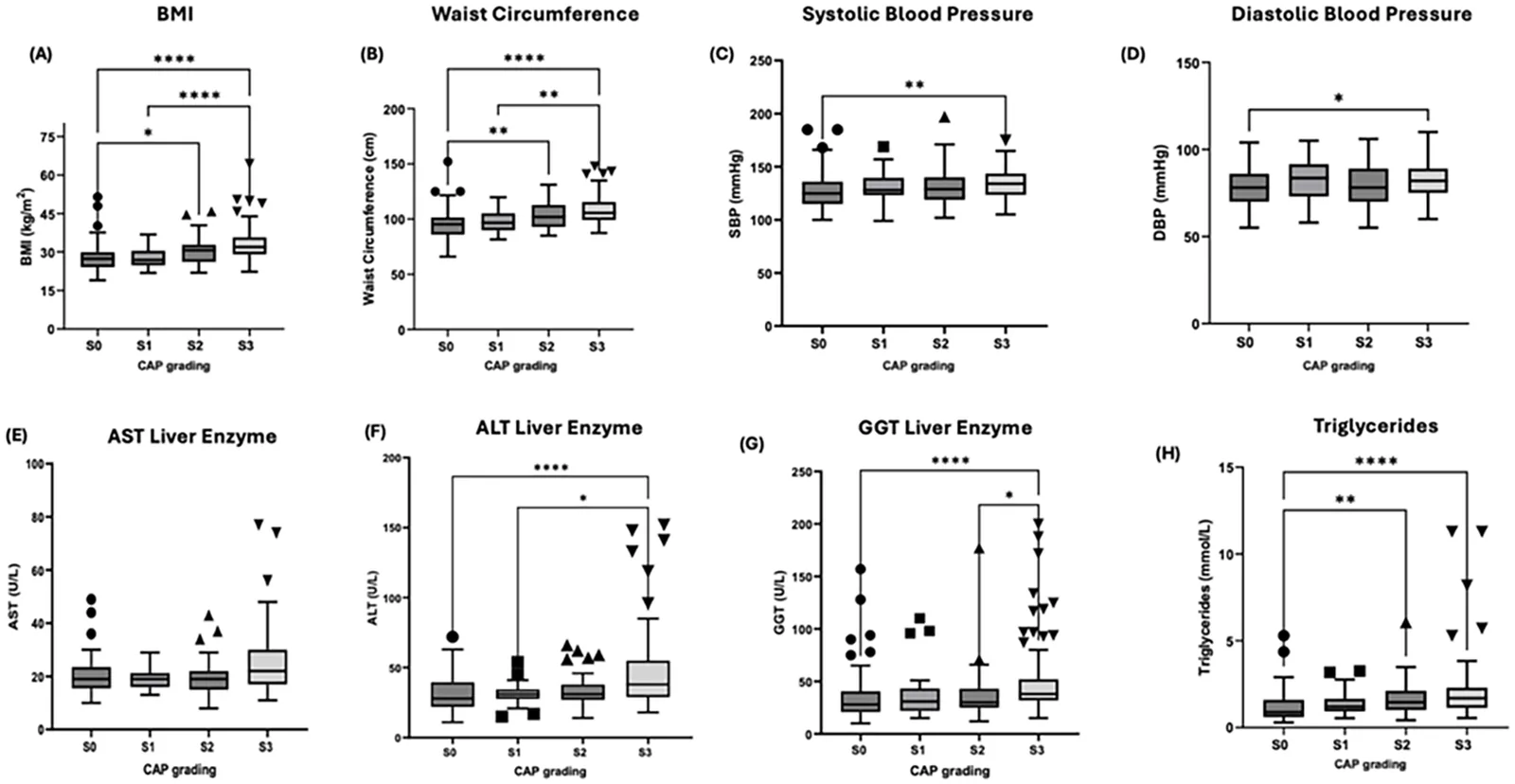
Clinical and metabolic characteristics stratified by hepatic steatosis grade. Box plots illustrates the distribution of **(A)** body mass index (BMI, kg/m2), **(B)** waist circumference (cm), **(C)** systolic blood pressure (SBP, mmHg), **(D)** diastolic blood pressure (DBP, mmHg), **(E)** aspartate aminotransferase (AST, U/L), **(F)** alanine aminotransferase (ALT, U/L), **(G)** gamma-glutamyl transferase (GGT, U/L), and **(H)** triglycerides (mmol/L) across hepatic steatosis grades. Each box represents the **(IQR)** with the horizontal line denoting the median. Whiskers extend to the furthest data points within 1.5 X IQR, and individuals beyond the whiskers represent outliers, group comparisons were performed using the Kruskal-Wallis test with Dunn’s *post-hoc* correction for multiple comparisons. *p < 0.05, **p < 0.01, ****p < 0.0001.

### 3.3 Fibrosis severity and associated clinical characteristics

The distribution of fibrosis grades is summarized in **Table 3** and **Figure 3**. The vast majority of participants had minimal fibrosis (F0-F1; 92.5%, n=247), while advanced fibrosis (F3-F4) was present in 3.4% of the cohort. Due to small sample sizes in F2-F4, these groups were combined for analysis (n=20). CAP score was significantly higher in the F2-F4 groups compared to F0-F1 (median 330 vs 272 dB/m; p<0.001; **Figure 3A**). BMI (median 33.4 vs 29 kg/m2; p<0.001; **Figure 3C**), weight (median 100.5 vs 83 kg; p<0.0001; **Figure 3F**), and waist circumference (median 120.5 vs 100.6 cm; p<0.0001; **Figure 3E**) were all significantly higher in participants with advanced fibrosis. GGT (p<0.05; **Figure 3B**) and serum creatinine (p<0.05; **Figure 3D**) were also significantly elevated in the F2-F4 group, collectively indicating a greater metabolic burden in those with advanced liver disease.

**Table 3:** Comparison of LSM across all groups, kPa score.

Parameters	F0-F1 (n=247)	F2 (n=11)	F3 (n=4)	F4 (n=5)	P-value
Number (%)	247 (92.5%)	11 (4.1%)	4 (1.5%)	5 (1.9%)	
Gender (M% F%)***	65.6/34.4	90.9/9.1	100.0/0.0	100.0/0.0	0.012
Age (in years), median (IQR)*	53.00 (45.00,60.25)	48.50 (44.00,58.50)	74.00 (68.00,75.00)	54.00 (53.50,65.50)	0.086
Height (cms), median (IQR)**	168.75 (161.00,176.00)	172.15 (162.53,176.25)	173.00 (168.00,173.50)	175.00 (174.25,176.00)	0.163
Weight (Kgs), Mean ± SD**	84.84 ± 16.64	117.75 ± 26.94	97.63 ± 12.70	115.64 ± 32.39	<0.001
BMI, kg/m^2^ median (IQR)*	29.12 (25.87,32.72)	38.75 (32.24,49.64)	35.76 (31.42,36.67)	30.77 (28.66,40.63)	<0.001
Waist Cir. (cm) Mean ± SD**	100.68 ± 12.28	125.15 ± 14.59	114.50 ± 11.62	126.04 ± 20.35	<0.001
SBP, mmHg, median (IQR)*	130.00 (120.00,140.00)	135.50 (120.75,156.00)	128.00 (125.00,147.00)	134.00 (134.00,140.50)	0.204
DBP, mmHg, median (IQR)*	80.00 (72.75,88.00)	87.50 (80.00,100.75)	67.00 (63.00,79.50)	90.00 (84.50,93.00)	0.026
S.Creat., umol/L, median (IQR)*	72.00 (60.75,82.00)	85.50 (69.50,95.25)	107.00 (101.50,114.00)	72.00 (64.50,78.50)	0.010
ALT, U/L, median (IQR)*	32.00 (27.00,44.00)	37.00 (31.75,53.25)	24.00 (23.50,25.00)	40.00 (37.50,54.50)	0.331
AST, U/L, median (IQR)*	20.00 (16.00,24.00)	27.00 (18.75,33.25)	19.00 (17.00,20.00)	23.00 (19.00,32.50)	0.085
GGT, U/L, median (IQR)*	35.00 (25.00,43.25)	47.00 (35.50,59.50)	29.00 (22.00,36.00)	97.00 (63.00,137.00)	0.016
TC, mmol/L, Mean ± SD **	4.77 ± 1.26	5.21 ± 1.10	4.15 ± 0.87	5.46 ± 2.01	0.302
TGL, mmol/L, median (IQR)*	1.32 (0.88,1.89)	1.75 (1.09,2.23)	1.22 (1.10,1.42)	1.55 (1.10,1.91)	0.270
HDL-C, mmol/L, median (IQR)*	1.29 (1.10,1.58)	1.13 (1.00,1.44)	1.04 (0.93,1.16)	1.28 (1.06,1.32)	0.089
LDL-C, mmol/L, median (IQR)*	2.75 (1.90,3.50)	3.15 (2.43,3.93)	2.10 (1.95,2.95)	3.40 (2.90,4.85)	0.593
HbA1C, %, median (IQR)*	6.70 (5.90,7.73)	6.70 (5.75,7.73)	6.90 (6.35,9.10)	5.70 (5.70,8.45)	0.818
Platelet x 10^9^/L, median (IQR)*	268.00 (229.00,299.25)	279.50 (194.50,329.50)	269.00 (264.50,274.00)	204.00 (136.00,263.50)	0.182
CAP Score, dB/m, median (IQR)	272.00 (230.75,313.00)	345.00 (318.00,376.25)	231.00 (230.00,272.00)	313.00 (300.00,336.00)	0.001

IQR, Inter quartile range; BMI, Body Mass Index; SBP, Systolic Blood Pressure; DBP, Diastolic Blood Pressure; S.Creat., Serum Creatinine; ALT, Alanine Transaminase; AST, Aspartate aminotransferase; GGT, gamma-glutamyl transferase; TC, Total Cholesterol; TGL, Triglycerides; HDL-C; high-density lipoprotein cholesterol; LDL-C, low-density lipoprotein cholesterol, CAP, Controlled Attenuation Parameter. *P-value continuous variable non-normal using Kruskal Wallis Test, **P-Value using parametric One-Way ANOVA, ***Fisher’s exact Test.

**Figure 3:**
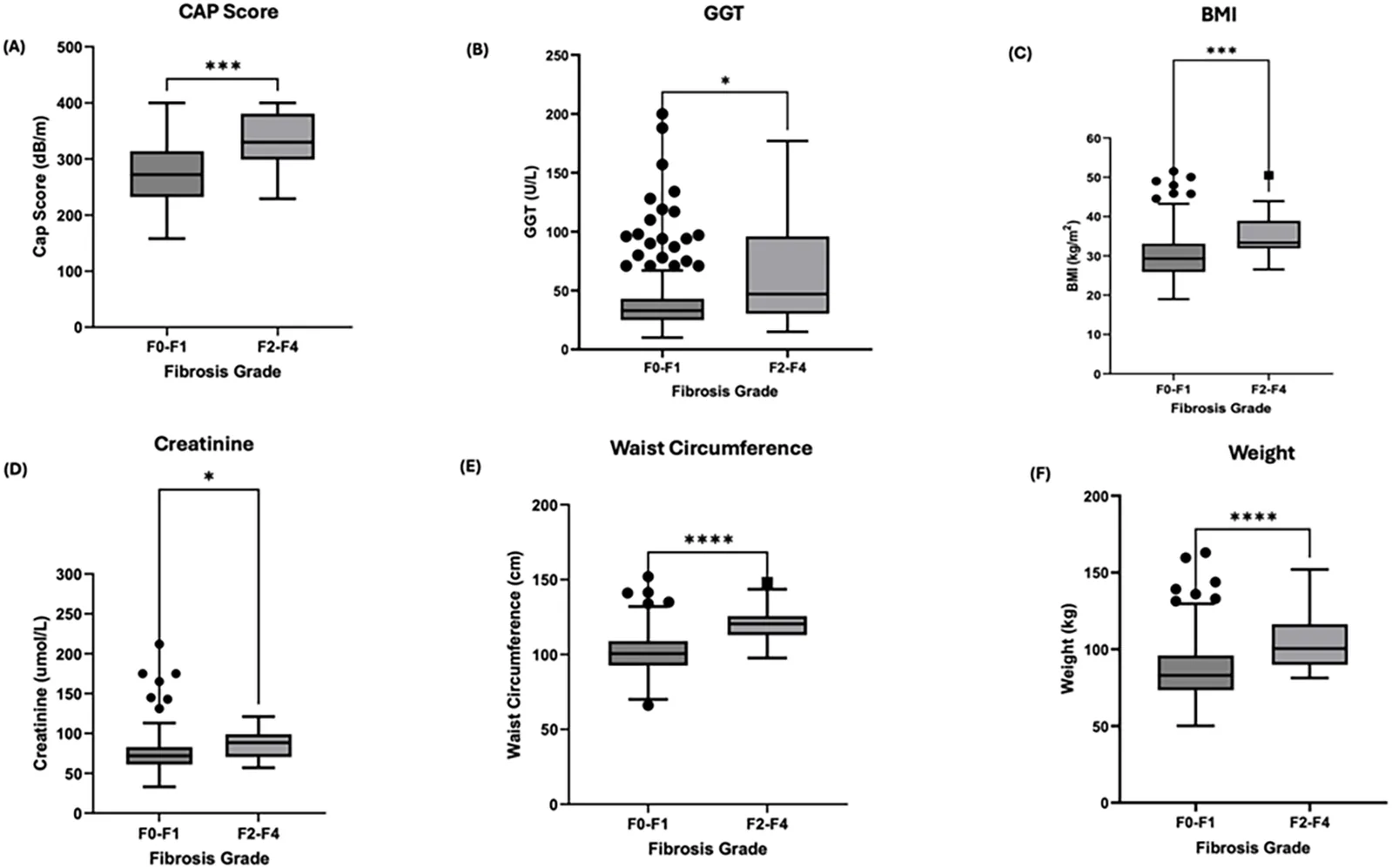
Clinical and metabolic characteristics stratified by fibrosis grade. Box plots illustrate the distribution of **(A)** FibroScan controlled attenuation parameter (CAP) score (dB/m), **(B)** (GGT, U/L), **(C)** (BMI, kg/m2), **(D)** serum creatinine (µmol/L), **(E)** waist circumference (cm), and **(F)** weight (kg) compared between participants with minimal fibrosis (F0-F1, LSM < 7.5 kPa, n=247) and significant fibrosis (F2-F4, LSM ≥ 7.5 kPa, n=20). F2-F4 groups were combined for analysis due to small sample sizes.

### 3.4 Bivariate and partial correlation analyses

Spearman correlation coefficients, adjusted for age and BMI (**Table 4**), revealed that CAP score was positively correlated with waist circumference (ρ = 0.200, p = 0.001), TG (ρ = 0.310, p < 0.001), ALT (ρ = 0.325, p < 0.001), AST (ρ = 0.197, p = 0.002), and GGT (ρ = 0.203, p = 0.001). CAP score correlated inversely with HDL-C (ρ = –0.186, p = 0.003). Fibrosis score significantly correlated with waist circumference, weight, ALT, AST, GGT, and platelet count (all p < 0.05). A modest but significant correlation was observed between CAP and LSM (ρ = 0.156, p = 0.013).

**Table 4:** Spearman correlation for steatosis CAP score and LSM kPa score adjusted age and BMI.

	Steatosis CAP Score		LSM kPa Score	
Parameters	Spearman ρ	P-value	Spearman ρ	P-value
Height (cms)	0.109	0.083	0.168	0.007
Weight (kgs)	0.103	0.102	0.193	0.002
Waist Circum (cms)	0.200	0.001	0.229	<0.001
SBP mmHg	0.044	0.490	0.039	0.541
DBP mmHg	0.145	0.021	0.102	0.107
S.Creat umol/L	-0.026	0.678	0.016	0.803
ALT U/L	0.325	<0.001	0.150	0.017
AST U/L	0.197	0.002	0.162	0.010
GGT U/L	0.203	0.001	0.296	<0.001
TC mmol/L	0.200	0.001	0.104	0.099
TG mmol/L	0.310	<0.001	0.078	0.215
HDL-C mmol/L	-0.186	0.003	-0.087	0.167
LDL-C mmol/L	0.193	0.002	0.118	0.062
HBA1C %	0.108	0.087	0.016	0.795
Platelet 109/L	0.066	0.299	-0.140	0.026
CAP Score			0.156	0.013
LSM, kPa	0.156	0.013		

SBP, Systolic Blood Pressure; DBP, Diastolic Blood Pressure; S.Creat., Serum Creatinine; ALT, Alanine Transaminase; AST, Aspartate aminotransferase; GGT, gamma-glutamyl transferase; TC, Total Cholesterol; TGL, Triglycerides; HDL-C; high-density lipoprotein cholesterol; LDL-C, low-density lipoprotein cholesterol, CAP, Controlled Attenuation Parameter.

### 3.5 Multivariable linear regression modeling

Multivariable linear regression analyses (**Table 5**) demonstrated that when evaluating steatosis severity (Model 1, R2 = 0.28) the CAP score was independently associated with liver stiffness measurement (LSM), BMI, ALT, and AST. Conversely, when modeling fibrosis severity (Model 2, R2 = 0.21) LSM was independently predicted by CAP score, BMI, ALT, AST, and GGT. Collectively, these findings indicate a close relationship between hepatic steatosis, fibrosis, and metabolic and hepatic injury markers in adults with T2DM, without implying causality given the cross-sectional study design. Although AST was positively correlated with CAP in the bivariate analysis, its coefficient became negative following multivariable adjustment. Similarly, ALT showed a negative adjusted coefficient in the LSM model despite its positive bivariate association with LSM. VIF values for ALT (3.76) and AST (3.26) were below 5, indicating no problematic multicollinearity. The changes in coefficient direction likely reflect shared variance between these closely related hepatic enzymes and a statistical suppression effect resulting from their simultaneous inclusion in the multivariable models. Accordingly, these negative coefficients should not be interpreted as evidence of inverse biological associations. Additional analyses were performed to evaluate the potential influence of diabetes pharmacotherapy on the observed associations. Baseline treatment distributions across CAP-defined steatosis categories are presented in **Supplementary Table 1**. After adjustment for available treatment categories, BMI, ALT, and LSM remained significantly and positively associated with CAP score (all p ≤ 0.027). Similarly, CAP, BMI, ALT, AST, and GGT remained independently associated with LSM (all p ≤ 0.027). Insulin therapy, metformin monotherapy, and multidrug therapy were not independently associated with either CAP or LSM in the adjusted models (**Supplementary Table 2**).

**Table 5:** Multiple linear regression for the association between steatosis CAP scores & LSM (kPa).

	Independent variable	β (SE)	95% CI	p-value	R^2^
Model 1 Dependent variable: steatosis CAP Score	LSM, kPa	3.28 (1.42)	0.48 – 6.09	0.022	0.28
	Age (in years)	0.32 (0.26)	-0.19 – 0.82	0.223	
	BMI, kg/m^2^	3.26 (0.52)	2.24 – 4.28	<0.001	
	ALT, U/L	1.20 (0.29)	0.64 – 1.76	<0.001	
	AST, U/L	-1.20 (0.59)	-2.37 - -0.03	0.045	
	GGT, U/L	0.02 (0.13)	-0.23 – 0.28	0.864	
Model 2 Dependent variable: LSM (kPa)	CAP Score	0.01 (0.00)	0.00 – 0.01	0.022	0.21
	Age (in years)	0.00 (0.01)	-0.02 – 0.02	0.789	
	BMI, kg/m^2^	0.07 (0.02)	0.02 – 0.12	0.004	
	ALT, U/L	-0.04 (0.01)	-0.06 - -0.01	0.003	
	AST, U/L	0.07 (0.03)	0.02 – 0.12	0.008	
	GGT, U/L	0.03 (0.01)	0.02 – 0.04	<0.001	

Values are unstandardized regression coefficients (β) with standard errors (SE) and 95% confidence intervals (CI). BMI, Body Mass Index; ALT, alanine aminotransferase; AST, aspartate aminotransferase; GGT, gamma-glutamyl transferase.

## 4 Discussion

MASLD is highly prevalent in the Middle East and among individuals with T2DM; however, data characterizing its prevalence and steatosis severity in Kuwaiti patients with T2DM remain limited. In this studied Kuwaiti cohort, 72.7% of patients with T2DM exhibited hepatic steatosis, underscoring the substantial burden of MASLD among adults with T2DM in Kuwait. This finding aligns with previous reports indicating that approximately 67% of patients with T2DM have hepatic steatosis, placing them at increased risk for hepatocellular carcinoma and liver-related mortality (13, 14). The observed prevalence of hepatic steatosis (72.7%) is also consistent with the substantial burden of MASLD reported across the MENA region. A recent systematic review and meta-regression analysis of data from 21 MENA countries estimated the prevalence of NAFLD/MASLD at 39.43% in the general population and 68.71% among patients with T2DM, with Kuwait predicted to have the highest MASLD prevalence at 45.37% (6). our observed prevalence of 72.7% in Kuwaiti T2DM patients is notably higher than the MENA pooled estimate of 68.71% for T2DM patients (6). This difference may reflect the severe metabolic burden in our cohort, characterized by a median BMI of 29.3-31.9kg/m2 across steatosis grades, and high rates of dyslipidemia and hypertension. Steatosis severity was strongly associated with key metabolic and hepatic parameters, including BMI, waist circumference, triglycerides, and liver enzymes (ALT, AST, and GGT), reinforcing the close link between metabolic dysregulation and liver fat accumulation. In addition, steatosis severity was associated with higher blood pressure and increased liver stiffness, indicating a progressive relationship with hypertension and fibrosis risk. Importantly, patients with advanced fibrosis displayed a more adverse metabolic profile, characterized by greater adiposity and elevated GGT and serum creatinine levels, further emphasizing the interplay between obesity, metabolic dysfunction, and liver disease progression. Collectively, these findings underscore the high burden and clinical complexity of MASLD in T2DM and highlight the need for integrated, risk-based screening and management strategies in this high-risk population.

The median age was 54 years, with minimal variation across steatosis grades (53–55 years). Although the difference was statistically significant (p = 0.044), the small 2-year variation is unlikely to be clinically meaningful. The mean age of our cohort (53.2 ± 9.8 years) is consistent with published data from similar clinical populations in the MENA region and gulf states. The SOLID registry, a national multicenter NAFLD database from Saudi Arabia representing one of the largest MENA region cohorts, reported a median age of 42.6 ± 13.6 years among NAFLD patients (15), while FibroScan-based T2DM study from Iraq reported a mean age of 47.7 ± 8.3 years (16), suggesting that MASLD manifests at a relatively young age in the MENA region, and that the median age of 54 years in our Kuwaiti T2DM cohort in consistent with this regional pattern. Notably, a FibroScan-based study of Emirati T2DM patients, reported that steatosis and fibrosis severity were unaffected by age, a finding consistent with our own observation (17). Furthermore, partial Spearman correlation analysis showed that age was not an independent predictor of CAP score or LSM after adjustment for BMI, liver enzymes, and FibroScan parameters, suggesting that metabolic and hepatic factors may have a greater influence on liver disease severity in this T2DM cohort. The observed findings should also be considered in the context of the established relationship between age and MASLD. Previous studies have reported that the prevalence of MASLD increases with advancing age in the general population, with a particularly pronounced increase among men over 50 years of age (11, 18). However, among individuals with established T2DM, the substantial metabolic burden associated with the disease may exert a stronger influence on hepatic manifestations than age alone. In the present cohort, steatosis was more frequently observed in males (67.8%) than females (32.2%); however, sex distribution did not differ significantly across steatosis grades (p = 0.371). This lack of a significant association may partly reflect the different age-related trajectories of MASLD between men and women, as the prevalence of MASLD in women tends to increase after menopause (19, 20). Interestingly, HbA1c did not differ significantly across steatosis grades despite the clear associations of CAP with adiposity, triglycerides, and liver enzymes. HbA1c primarily reflects glycemic exposure over the preceding several months and may not fully capture the longer-term metabolic disturbances contributing to hepatic fat accumulation. Furthermore, glycemic control in individuals with established T2DM may be influenced by factors such as diabetes duration and glucose-lowering therapy. Thus, the severity of hepatic steatosis in this cohort may be more closely related to the broader metabolic phenotype, including adiposity, insulin resistance, and dyslipidemia, than to contemporaneous HbA1c levels alone.

Overall, these findings underscore the substantial burden of MASLD among Kuwaiti patients with T2DM and indicate that demographic factors such as age and sex should be interpreted alongside metabolic and hepatic risk factors rather than considered in isolation. The results further support the need for risk-stratification approaches that account for sex- and age-related differences while prioritizing the metabolic characteristics that may more strongly influence the progression of liver disease in patients with T2DM. Additionally, steatosis severity showed significant positive associations with BMI, waist circumference, and serum TGs, consistent with numerous previous reports (21, 22). Recent studies have shown a positive correlation between ALT and AST levels and the severity of steatosis (23, 24). Similarly, GGT levels were positively associated with steatosis severity, aligning with prior evidence demonstrating a relationship between elevated GGT and hepatic steatosis (25). Collectively, these findings further support the close interplay between metabolic dysregulation and hepatic steatosis in patients with T2DM.

Both SBP and DBP differed significantly across steatosis grades, indicating a clear association between MASLD and hypertension in patients with T2DM. Notably, SBP showed a progressive increase from 125mmHg in S0 to 134mmHg in S3, while DBP demonstrated a non-linear pattern without clear directional trend. This pattern may reflect the greater contribution of arterial stiffness, rather than peripheral vascular resistance as the primary hypertensive mechanism in this cohort, although direct hemodynamic assessment would be required to confirm the dominant mechanism. Evidence supports that individuals with MASLD exhibit increased arterial stiffness linked to shared metabolic pathogenesis (26, 27) and that arterial stiffening precedes isolated systolic hypertension in patients with T2DM (28). Similar associations between MASLD severity and elevated blood pressure have been reported in regional MENA populations, a study from Egypt demonstrated that steatosis and fibrosis severity were associated with increased SBP and DBP (29), while a Saudi Arabian T2DM cohort reported hypertension in 71.6% of patients with MASLD (30), underscoring the high cardiovascular burden accompanying MASLD in this region. The observation of clear association between MASLD and hypertension in patients with T2DM is consistent with recent evidence reporting that approximately 26% of individuals with MASLD have concomitant hypertension (31, 32). Median liver stiffness increased significantly from 4.5 kPa in S0 to 5.2 kPa in S3 (p <0.001), suggesting that greater steatosis severity is progressively associated with higher fibrosis risk in T2DM, consistent with previous reports (33–35).

Regarding fibrosis severity, the majority of participants had minimal fibrosis (94%), while advanced fibrosis was presented in only 3% of the cohort. Despite this relatively low prevalence of advanced fibrosis, participants with significant fibrosis (F2-F4) demonstrated a markedly more adverse metabolic profile compared to those with minimal fibrosis. Specifically, BMI, body weight, and waist circumference, were all significantly higher in groups F2-F4, highlighting the central role of obesity and visceral adiposity in fibrosis progression. GGT and serum creatinine were also significantly elevated in F2-F4 group. The elevation of serum creatinine in participants with significant fibrosis in our cohort is consistent with a growing body of evidence establishing a bidirectional relationship between hepatic fibrosis severity and renal dysfunction in MASLD. MASLD is recognized as a systemic metabolic disorder with extrahepatic organ involvement, and chronic kidney disease (CKD) represents one of its most clinical manifestations (36, 37). In a landmark nationwide multicenter study of 10,873 MASLD patients from Türkiye, hepatic fibrosis severity assessed by FIB-4 index was independently associated with reduced eGFR, with patients in the high FIB-4 group having significantly lower eGFR than those with low FIB-4, after adjustment for diabetes, hypertension, BMI, and SGLT2 inhibitor use (36). Further supporting this association, previous studies reported positive associations between serum creatinine levels and fibrosis severity in MASLD (38, 39), collectively reinforcing the clinical significance of monitoring renal markers in the contact of hepatic fibrosis. This co-existence of hepatic fibrosis and reduced kidney function likely reflects shared pathophysiological mechanisms including insulin resistance, chronic low-grade inflammation, oxidative stress, and renin-angiotensin-aldosterone system activation, which simultaneously drive fibrogenesis in the liver and nephrosclerosis in the kidney (40). Furthermore, CAP score was significantly higher in participants with significant fibrosis, reinforcing the bidirectional relationship between hepatic steatosis and fibrosis progression. Collectively, these findings highlight the complex interplay between hepatic steatosis, fibrosis progression, and systemic metabolic impairment in adults with T2DM. Importantly, the principal associations with CAP and LSM remained significant after additional adjustment for available diabetes treatment regimens, suggesting that these findings were not substantially altered by the measured pharmacotherapy categories. Nevertheless, detailed information on all potentially relevant medications, including statin therapy, was not available. Residual confounding from specific glucose-lowering or lipid-lowering agents therefore cannot be excluded. The single-center design should be considered when interpreting the generalizability of these findings. Participants were recruited from a specialized diabetes center and may therefore differ from the broader population of individuals with T2DM in Kuwait in terms of clinical characteristics, metabolic risk profile, and healthcare utilization. Consequently, the prevalence estimates reported in this study should be interpreted as reflecting the studied Kuwaiti T2DM clinical cohort rather than national population-level estimates. Future multicenter and population-based studies would be valuable to determine the broader prevalence and severity of MASLD among individuals with T2DM in Kuwait.

## 5 Conclusion

In conclusion, this cross-sectional study provides a comprehensive assessment of MASLD prevalence and steatosis severity among patients with T2DM in Kuwait. Hepatic steatosis (S1–S3) was identified in 72.7% of the cohort, with 40.8% classified as having severe steatosis (S3). Steatosis severity was positively associated with BMI, waist circumference, triglycerides, and liver enzymes (ALT, AST, and GGT), reflecting a close link between hepatic involvement and metabolic dysregulation. These findings offer robust, region-specific evidence supporting the integration of routine liver assessment into comprehensive diabetes care in Kuwait. Early identification and targeted management of MASLD in this high-risk population may help prevent progression to advanced liver disease, reduce liver-related morbidity and mortality, and lessen the long-term healthcare burden.

## Data Availability

The original contributions presented in the study are included in the article/**Supplementary Material**. Further inquiries can be directed to the corresponding authors.

## Supplementary material

Supplementary Table 1 Baseline pharmacotherapy breakdowns across steatosis tiers. No treatment data available considered as Diet + Lifestyle; * Combined and analyzed as Multidrug for Multiple linear regression.

Supplementary Table 2 Multiple Linear Regression for the association between Steatosis CAP Scores & LSM (kPa) after adjusting for treatments. Model 2 Adjusted for pharmacotherapy breakdowns; statin data not available
