# Correction: Prevalence and steatosis severity of MASLD in patients with type 2 diabetes mellitus in Kuwait

**DOI:** 10.3389/fendo.2026.2032618

**Published:** 2026-10-27

**Authors:** Munira Altarrah, Rashed Alhammad, Saoud Ahmad, Mohamed Abu-Farha, Sriraman Devarajan, Shaymaa Alhaddad, Abeer Afyoni, Fouz AlHaifi, Mohamed Qaddoumi, Mohammad Shehab, Mohamed Salah, Hamad Ali, Muhammed Abdul Ghani, Fahd Al-Mulla, Fahad Alajmi, Jehad Abubaker

**Affiliations:** 1 Unit of Gastroenterology and Transplant Hepatology, Thunayan Al Ghanim Gastroenterology Center, Al Amiri Hospital, Kuwait City, Kuwait; 2 Department of Pharmacology, Faculty of Medicine, Kuwait University, Kuwait City, Kuwait; 3 Department of Translational Research, Dasman Diabetes Institute, Kuwait City, Kuwait; 4 Department of Biochemistry and Molecular Biology, Dasman Diabetes Institute, Kuwait City, Kuwait; 5 National Dasman Diabetes Biobank, Dasman Diabetes Institute, Kuwait City, Kuwait; 6 Department of Pharmacology, Faculty of Pharmacy, Kuwait University, Kuwait City, Kuwait; 7 Program of Medicine, College of Medicine and Health Sciences, Abdullah Al Salem University, Khaldiya, Kuwait; 8 Department of Medical Laboratory Sciences, Faculty of Allied Health Sciences, Health Sciences Center, Kuwait University, Jabriya, Kuwait; 9 Division of Diabetes, University of Texas Health Science Center, San Antonio, TX, United States

**Keywords:** fatty liver, MASLD, metabolic disease, steatosis, type 2 diabetes

There was a mistake in the author name “Mohamed Shehab”. The correct name is “Mohammad Shehab”.

Affiliation “Program of Medicine, College of Medicine and Health Sciences, Abdullah Al Salem University, Khaldiya, Kuwait” was omitted from the paper. This affiliation has now been added for author “Mohammad Shehab”.

The original version of this article has been updated.

