# Supplementary material for: Prevalence and steatosis severity of MASLD in patients with type 2 diabetes mellitus in Kuwait
Source: Front Endocrinol (Lausanne). 2026 Oct 13;17:1963647. doi: 10.3389/fendo.2026.1963647 (PMC13710078; doi:10.3389/fendo.2026.1963647)
Supplement: Supplementary file 1 [file SupplementaryFile1.docx]

Supplementary table 1. Baseline pharmacotherapy breakdowns across steatosis tiers.

| **Parameters** | ***S0* (no steatosis, <238 dB/m)** | ***S1* (mild steatosis, 238–259 dB/m)** | ***S2* (moderate steatosis, 260–290 dB/m)** | ***S3* (severe steatosis, >290 dB/m)** | **P-value** |
| --- | --- | --- | --- | --- | --- |
| **Number** | 73 (27.3%) | 30 (11.2%) | 55 (20.6%) | 109 (40.8%) |  |
| Insulin Yes / No | 5 | 2 | 3 | 2 | 0.319 |
| Metformin + SGLT2* | 8 | 1 | 3 | 5 | 0.331 |
| Metformin + SGLT2 + DPP4* | 9 | 3 | 7 | 9 | 0.633 |
| Metformin Alone | 2 | 4 | 7 | 4 | 0.038 |
| Diet + Lifestyle | 49 | 20 | 35 | 89 | 0.036 |

No treatment data available considered as Diet + Lifestyle; * Combined and analyzed as Multidrug for Multiple linear regression.

Supplementary table 2. Multiple Linear Regression for the association between Steatosis CAP Scores & LSM (kPa) after adjusting for treatments.

|  | Independent Variable | β (SE) | 95% CI | p-value | R^2^ |
| --- | --- | --- | --- | --- | --- |
| Model 1  Dependent variable: steatosis CAP Score adjusted for treatment | LSM, kPa | 3.14 (1.42) | 0.35 – 5.94 | 0.027 | 0.29 |
|  | Age (in years) | 0.29 (0.27) | -0.24 – 0.83 | 0.282 |  |
|  | BMI, kg/m^2^ | 3.16 (0.52) | 2.14 – 4.18 | <0.001 |  |
|  | ALT, U/L | 1.17 (0.29) | 0.60 – 1.73 | <0.001 |  |
|  | AST, U/L | -1.12 (0.60) | -2.29 - -0.06 | 0.063 |  |
|  | GGT, U/L | 0.02 (0.13) | -0.23 – 0.28 | 0.863 |  |
|  | Insulin therapy. | -24.35 (15.17) | -54.23 – 5.52 | 0.110 |  |
|  | Monotherapy | -0.38 (12.47) | -24.93 – 24.17 | 0.976 |  |
|  | Multidrug glucose-lowering therapy | -15.69 (8.25) | -31.92 – 0.55 | 0.058 |  |
| Model 2  Dependent variable:  LSM Score adjusted for treatment | CAP Score | 0.00 (0.00) | 0.00 – 0.01 | 0.027 | 0.21 |
|  | Age (in years) | 0.00 (0.01) | -0.02 – 0.03 | 0.636 |  |
|  | BMI, kg/m^2^ | 0.07 (0.02) | 0.02 – 0.12 | 0.004 |  |
|  | ALT, U/L | -0.04 (0.01) | -0.06 - -0.01 | 0.003 |  |
|  | AST, U/L | 0.07 (0.03) | 0.02 – 0.12 | 0.011 |  |
|  | GGT, U/L | 0.03 (0.00) | 0.02 – 0.04 | <0.001 |  |
|  | Insulin therapy | 0.24 (0.66) | -1.07 – 1.55 | 0.716 |  |
|  | Monotherapy | 0.01 (0.54) | -1.07 – 1.08 | 0.987 |  |
|  | Multidrug glucose-lowering therapy | -0.25 (0.36) | -0.96 – 0.47 | 0.496 |  |

Model 2 Adjusted for pharmacotherapy breakdowns; statin data not available
